# Cold Plasma Treatment of Sunflower Seeds Modulates Plant-Associated Microbiome and Stimulates Root and Lateral Organ Growth

**DOI:** 10.3389/fpls.2020.568924

**Published:** 2020-08-28

**Authors:** Inga Tamošiūnė, Dalia Gelvonauskienė, Perttu Haimi, Vida Mildažienė, Kazunori Koga, Masaharu Shiratani, Danas Baniulis

**Affiliations:** ^1^Institute of Horticulture, Lithuanian Research Centre for Agriculture and Forestry, Babtai, Lithuania; ^2^Faculty of Natural Sciences, Vytautas Magnus University, Kaunas, Lithuania; ^3^Faculty of Information Science and Electrical Engineering, Kyushu University, Fukuoka, Japan; ^4^Center for Novel Science Initiatives, National Institutes of Natural Sciences, Tokyo, Japan

**Keywords:** 16S RNA sequencing, cold plasma, metagenomics, proteomics, seed, stress response

## Abstract

Cold atmospheric pressure (CP) plasma irradiation of seeds has been shown to promote plant growth, but the molecular basis of this phenomenon is poorly understood. In our study, optimum irradiation of common sunflower seeds using a dielectric barrier discharge CP device stimulated growth of sunflower lateral organs and roots by 9–14% compared to the control. Metagenomic analysis revealed that the structure of plant-associated bacterial assembly was greatly modified upon CP treatment and could be attributed to the antimicrobial effect of CP-generated reactive species. The treatment resulted in the domination of spore forming *Mycobacterium* sp. in the above-ground tissues of the seedlings. While the overall bacterial diversity in the roots was barely affected, the CP-induced shift in microbial composition is the likely basis for the observed seedling root growth stimulation and the long-term effect on lateral organ growth and could be mediated by increase in water uptake and/or direct root signaling. Low amplitude protein abundance differences were detected in the roots of the emerging seedlings that are characteristic to low intensity stress stimuli response and could be linked to the changes in plant-associated microbiome upon CP treatment.

## Introduction

Plant seed treatment with high-frequency atmospheric-pressure plasma, also known as cold plasma (CP), has been shown to have a potential to enhance agronomic seed quality by surface decontamination, germination enhancement, and promoting plant growth (reviewed by Ohta (Ohta, 2016). The physical impact of high energy electrons, high frequency electromagnetic and UV radiation could have a direct impact on biological systems; however the main biologically active constituent of CP is a complex mixture of activated radical species produced by dissociation, excitation and ionization of gas atoms and molecules [mainly molecular oxygen (O_2_), nitrogen (N_2_) and water (H_2_O) in air atmosphere] upon collision with high energy electrons and in subsequent chemical reactions (Turner, 2016; Whitehead, 2016). At relatively low gas temperature and electron energies (2-5 eV), which are characteristic to CP, excitation of N_2_ molecules and dissociation of O_2_ in air atmosphere leads to accumulation of ozone (O_3_) (Whitehead, 2016). Higher electron energies lead to the dissociation of N_2_ and the production of nitrogen oxides (NO_x_) which inhibit ozone production, and subsequently recombine to form a number of other reactive nitrogen species including oxidant and nitrating agent peroxynitrite anion (ONOO^–^). Air humidity is also an important factor modulating output of the CP-generated reactive species. In the presence of water vapor, a hydroxyl radical (OH**^·^**) is produced in the primary electron collision process involving dissociation of water or by secondary process involving neutralization of ions and by reactions of excited states of O_2_ and N_2_ (Whitehead, 2016). Due to the presence of an unpaired electron, OH**^·^** radical is highly unstable and could readily oxidize a range of organic compounds (Gligorovski et al., 2015).

CP-generated reactive oxygen and nitrogen species (ROS/RNS) are known to have a negative impact on biological systems and have been used as sterilizing agents to inactivate microorganisms. Disruption of the microbial cell walls, as well as damage to the cell membrane, DNA and protein integrity and function has been reported for free-living and biofilm forming bacteria exposed to CP [reviewed by (Patil et al., 2016)]. Han et al. (Han et al., 2016) have proposed different mechanisms for the inactivation of gram- positive and negative bacteria that are linked to the impairment of the cell wall and the damage of intracellular components, respectively. CP treatment has been shown to effectively disrupt bacterial spores (Hong et al., 2009; Reineke et al., 2015). In addition to reactive chemical species, a negative effect of the UV irradiation on microorganisms and spores has been documented under certain conditions (Moreau et al., 2000; Moisan et al., 2001; Boudam et al., 2006), but its role remains uncertain (Patil et al., 2016). On the other hand, the effects of plasma depend on the dose and the composition of the reactive species generated; and the vitality and activity of plant growth-promoting bacteria enhancing effect following CP treatment has been reported (Ji et al., 2019).

An assembly of commensal and pathogenic microorganisms are passed through seeds and these are important for the survival and vigor of the germinated seedlings and plants (Nelson, 2018). Recently, metagenomic analysis of the bacterial *16S rRNA* gene or *gyrB* gene-based species-specific bacterial marker, as well as the fungal internal transcribed spacer, using next-generation sequencing (NGS) has proven to be a powerful technique to study the composition and the dynamics of plant-associated microbial communities (Tamosiune et al., 2019), and seed microbiomes have been investigated extensively (Barret et al., 2015; Klaedtke et al., 2016). Recently, metagenomic analysis revealed a selective effect on the composition of *Arabidopsis thaliana* seedling and mature plant microbiota by CP treatment of seeds (Tamosiune et al., 2020). In addition, the effect of CP on seed microbiome is supported by studies based on traditional microbiological techniques [reviewed by (Ohta, 2016)]. Efficient inactivation of native microflora upon atmospheric pressure CP treatment was reported for chickpea (Mitra et al., 2014), alfalfa, onion, radish, cress (Butscher et al., 2016a) and lentil seeds (Waskow et al., 2018). Another study used low pressure CP in air or sulfur hexafluoride atmosphere for a variety of cultivated plant seeds and demonstrated that CP effectively inactivates pathogenic strains of *Aspergillus* spp. and *Penicillum* spp. depending on seed surface, plasma gas type, plasma treatment time, and the microbial population density (Selcuk et al., 2008). Effective inactivation of an indicator strain of spore-forming *Bacillus atrophaeus* on *Brassica napus* seeds was achieved by direct application of dielectric barrier discharge (DBD) plasma (Schnabel et al., 2012). However, *Geobacillus stearothermophilus* endospore inactivation on wheat grains was less efficient and required extended treatment (Butscher et al., 2016b). This is presumed to result from complex surface structure and geometry of grains, where microorganisms can be sheltered by an uneven surface (Schnabel et al., 2012; Butscher et al., 2016b), and inactivation efficiency is dependent on substrate moisture level and plasma supply settings that determine outcome of treatment with reactive species (Butscher et al., 2016a).

Certain CP-generated ROS/RNS, such as superoxide anion (^-·^O_2_), NO, hydrogen peroxide (H_2_O_2_) or ONOO^–^, play an important role as signaling messengers in eukaryotic cells (Droge, 2002) and are implicated in the regulation of seed dormancy, germination (Jeevan Kumar et al., 2015) and plant physiology (Del Rio, 2015). Several studies have established that CP treatment of seeds results in a long-term growth enhancement of *Arabidopsis* (Koga et al., 2015), pea (Stolarik et al., 2015), radish (Kitazaki et al., 2014), soybean (Ling et al., 2014), sunflower (Mildaziene et al., 2019; Zukiene et al., 2019), wheat (Jiafeng et al., 2014), and Norway spruce (Pauzaite et al., 2017). CP treatment significantly increased the length and weight of maize roots (Henselova et al., 2012). Also, improved disease resistance was reported for tomato (Jiang et al., 2014). While the mechanism of CP-generated ROS/RNS action on plants is poorly understood, it is presumed that the response of plants to seed treatment is a consequence of stressor-induced eustress phenomena. Plants can produce more than one phenotype in different environments, and stress factors could have an effect on plant ontogenetic development throughout their life cycles (Dalal et al., 2017; Ibanez et al., 2017). Also, it is important to consider that stressor-induced plant physiology changes might affect plant colonization by seed and environment derived microorganisms leading to changes in plant-associated microbial community, and such changes may have an impact on plant growth.

Our earlier study using a low-pressure capacitively coupled CP device revealed that protein expression changes in common sunflower (*Helianthus annuus* L.) seedlings induced by pre-sowing plasma treatment of seeds were very similar to the effect exerted by electromagnetic field treatment, suggesting that electromagnetic field constituent of CP may induce the observed changes on plant physiology (Mildaziene et al., 2019). DBD devices operate at the ambient atmosphere and plasma discharge generates more complex and enriched ensemble of reactive O_2_ and N_2_ species of which many could have an impact on biological systems, such as antimicrobial effect or interference with signal transduction in seed tissues. Therefore the objectives of this study were to a) assess the effect of pre-sowing seed treatments with DBD plasma on development of common sunflower, b) to study the influence on composition of plant-associated bacteria, and c) to assess the change in protein expression pattern in roots. The effects of pre-sowing seed treatments on the main features of organ development, such as root length and leaf and inflorescence size, were assessed throughout the life-span of sunflower plants. As plasma treatment could have direct or indirect effect on plant-associated microbiome and its role in plasma stressor-induced plant response has been poorly understood, a metagenomic analysis of *16S rRNA* gene was used to analyze changes in the composition of plant-associated bacteria in seedlings of sunflower upon growth-stimulating treatment with CP. Proteomics analysis was used to study changes in protein expression patterns in roots of emerging seedlings to uncover protein expression patterns that might be linked to the direct plant response to CP and/or changes in the composition of plant-associated microbiome.

## Materials and Methods

### Pre-Sowing Seed Treatment With CP

Seeds of the common sunflower confectionery variety ‘Nyķrségi fekete’ harvested in 2016 were received from the Institutes for Agricultural Research and Educational Farm, University of Debrecen (Hungary). Seeds were stored at 4–10°C and were used for experiments carried out in 2018-19.

Seed treatment was carried out using a dielectric barrier discharge (DBD) device described by Koga et al., (2015). CP source (40 mm × 44 mm) constructed from 20 ceramic-coated stainless steel electrodes was used, and eight seeds per treatment were placed on a glass plate in an area of 20 mm × 30 mm in size that was expected to have a homogenous distribution of the discharge (estimated based on pH changes in a 96-well plate filled with bromophenol blue solution). The distance from the seed surface to the source of CP was maintained at approx. 3 mm. The discharge voltage, frequency and power were 7.0 kV, 14.4 kHz, and 4.64 W, respectively. Irradiation with CP was carried out at room temperature, and relative humidity was maintained at 40%–60%. To maintain seed surface temperature below 45°C, repetitive 2 min irradiation with 1 min rest intervals was applied up to 4 times to achieve cumulative CP irradiation of 2 min (CP2), 4 min (CP4), or 8 min (CP8). After CP treatment, seeds were stored in plastic bags for 1 week at 25°C in the dark. The control seeds were stored under the same conditions. Treatments for all experimental conditions were replicated 3 times.

### Measurement of Seedling and Plant Morphological Parameters

For morphology analysis at an early growth stage, seeds were sown in unsterile peat substrate and seedlings were maintained under controlled growth conditions in a climatic chamber under 16 h photoperiod at 22°C. Four days after seedling emergence, they were removed from substrate, roots were washed to remove the substrate, wiped with a paper towel, and the length of the roots and hypocotyls was estimated using image analysis software ImageJ (Schneider et al., 2012). During the first 2 weeks of growth, seedling height, length, and width of cotyledons and the first leaf was directly measured at 2-day intervals starting 1 week after seedling emergence.

For mature plant morphology analysis, plants were grown under field conditions. Plants from the three experimental groups and three replicates were randomly distributed as blocks of 8–10 plants in four separate rows. After 3 weeks, leaf length and width were measured and a cumulative value of leaf size was estimated based on the relative size of the first five leaves compared to the control group. The size of inflorescences was assessed after 3 months.

### Bacterial 16S RNA Metagenomic Analysis

Metagenomic analysis included samples of seedlings from the control and CP4 treated experimental groups. Samples of root and cotyledons were collected 4 days after seedling emergence and combined samples of leaves and cotyledons were collected after 2 weeks of cultivation under controlled growth conditions. Samples were flash frozen in liquid nitrogen, ground to fine powder and stored at -70°C.

Two different bacterial DNA extraction methods were employed and six unique libraries were created. Samples were prepared using the standard bacterial DNA purification method based on application of cetyltrimethylammonium bromide (CTAB) (Ding et al., 2013) (DNA extraction method 1) or extraction using sodium dodecyl sulfate (SDS) detergent (Wang et al., 2008) (DNA extraction method 2). All DNA extractions were performed according to the author instructions, with the exception of starter quantities adjusted to 2 g, and the final elution performed in 20 µl of nuclease free water.

DNA sequencing analysis was carried out using semiconductor-based Ion Torrent sequencing technology (Thermo-Fisher Scientific, USA). DNA library preparation procedure followed the 16S Metagenomic Sequencing Library Preparation Protocol (Thermo-Fisher Scientific, USA). The hypervariable regions of *16S rRNA* gene were amplified by two separate PCR reactions and V2–4–8, V3,6–7 specific primer sets of the Ion 16S Metagenomics Kit (Thermo-Fisher Scientific, USA). Cycling conditions were as follows: 25 cycles of 30 s denaturation at 95°C, 30 s annealing at 56°C and 20 s extension at 72°C. To confirm successful amplification of *16S rRNA* DNA samples, negative (water) and positive (*E. coli* DNA*)* controls were used. Equal volumes of V2–4–8 and V3,6–7 amplification reactions were combined. Final DNA library was made from 50 ng of combined amplicons with the Ion Plus Fragment Library Kit and Ion Xpress Barcodes Adapters, 1–16 (Thermo-Fisher Scientific, USA) following the manufacturer’s recommendations. The quality and concentration of the libraries were determined by the MCE-202 MultiNA DNA analyzer (Shimadzu, Japan). Adapter-ligated and nick-repaired DNA was purified using 1.4 volumes of Agencourt AMPure beads (Beckman Coulter, USA) and eluted in Tris-EDTA buffer. The accurate library concentration for template preparation was estimated by qPCR and Ion Library TaqMan Quantitation Kit (Thermo-Fisher Scientific, USA). Each sample was adjusted to 10 pM. Equal volumes of all samples were combined and emulsion PCR was carried out using Ion OneTouch 2 System and Ion PGM Hi-Q View OT2 Kit (Thermo-Fisher Scientific, USA). The amplified clonal libraries were enriched using Ion PGM Enrichment Beads on Ion OneTouch ES instrument (Thermo-Fisher Scientific, USA). The enrichment efficiency was assessed using Ion Sphere Quality Control Kit (Thermo-Fisher Scientific, USA). Prepared template spheres were loaded on Ion 316 v.2 chip and sequencing was performed on Ion Personal Genome Machine (PGM) system using Ion PGM Hi-Q Sequencing Kit (Thermo-Fisher Scientific, USA).

Base calling and run demultiplexing were performed by Torrent Suite v.5.0.5 (Thermo-Fisher Scientific, USA) with default parameters. Sequencing data was processed using 16S Metagenomic workflow of the Ion Reporter Software v.5.10.5.0 (Thermo-Fisher Scientific, USA). Reads were trimmed by primers at both ends. Threshold for unique reads was set to 10. Taxonomic identification was performed using MicroSEQ 16S Reference Library v.2013.1 and Greengenes v.13.5 databases. Threshold value for percentage identity for genus and species ID was 97%.

### Sunflower Seedling Proteome Analysis Using Two-Dimensional Electrophoresis

For proteome analysis of roots of emerging seedlings, the experimental design and sample preparation was the same as that for the bacterial metagenomic analysis. Total cell protein was prepared using phenol extraction and ammonium acetate precipitation, as described previously (Isaacson et al., 2006). Internal standards were prepared from a pooled mixture of all protein extracts. Protein separation and detection was performed using a differential gel electrophoresis procedure as described previously (Tamosiune et al., 2018). Sample aliquots of 50 µg were labeled with Cy3 and Cy5 fluorescent dyes, and the internal standard was labeled with Cy2 dye (Lumiprobe, USA). For the preparative gel, 500 µg of unlabeled internal standard was mixed with 50 µg of Cy2 labeled internal standard. Isoelectric focusing was performed on 24 cm IPG strips with a linear gradient of pH 4–7 using Ettan IPGphor (GE Healthcare, USA). Further, the proteins were separated on 1-mm thick 10-16% polyacrylamide gradient gels using Ettan DALTsix (GE Healthcare, USA). Gels were scanned using a fluorescence scanner FLA 9000 (GE Healthcare, USA). Relative protein quantification was performed using DeCyder 2-D Differential Analysis Software, v.7.0 (GE Healthcare, USA), and the Biological Variation Analysis module was used to match protein spots of four biological repeats and experimental groups.

Preparative gel was fixed in 50% methanol and 10% acetic acid. Protein spots were excised manually and subjected to protein digestion with trypsin, according to a method described previously (Shevchenko et al., 2006). Protein digests were loaded and desalted on a 100 μm × 20 mm Acclaim PepMap C18 trap column and separated on a 75 μm × 150 mm Acclaim PepMap C18 column using an Ultimate3000 RSLC system (Thermo-Scientific, USA), coupled to a Maxis G4 Q-TOF mass spectrometer detector with a Captive Spray nano-electrospray ionization source (Bruker Daltonics, Germany). Peptide identification was performed using the MASCOT server (Matrix Science, USA) against *Helianthus annuus* L., genome database v.1.0 (Badouin et al., 2017). Threshold value for the identification of proteins was a Mascot score of >50 and at least two peptides.

For protein annotation, NCBI Protein database was queried using Blast2GO software (Conesa et al., 2005). Arabidopsis homologues were identified by BLAST search against TAIR10 gene models at the Sunflower Genome Database (https://sunflowergenome.org/blast/). Protein interactions were assessed using the String database (Szklarczyk et al., 2015).

### Statistical Data Analysis

The seed treatment and seedling cultivation experiments were carried out 4–6 times to confirm reproducibility, and samples used for metagenomic and proteome analysis were collected from at least three separate experiments. Means of morphological parameters were compared between the experimental groups using one-way analysis of variance function of Prism v. 3 (GraphPad Software Inc.) and significance of differences compared to the control were identified using Tukey post-hoc analysis (p < 0.05). Data are presented as mean of at least 3 independent experiments and standard error of the mean.

For the 16S rRNA analysis, operational taxonomic units (OTU) abundance information was normalized to the sample with the fewest sequences and alpha diversity and beta diversity were subsequently performed. Four indices were applied to analyze Alpha diversity: observed-species, Chao1, Shannon and Simpson indices. These metrics reflected diversity and richness of the individual samples and were plotted using IonRerporter v.5.10.5.0 software. Beta diversity was used to compare diversity of bacterial communities among the six experimental groups. The bacterial richness at genus level was quantified using principal coordinate analysis (PCoA) and abundance data using Bray-Curtis metrics (IonRerporter v.5.10.5.0), and the relative bacterial species abundance was visualized using heatmap software (https://software.broadinstitute.org/morpheus).

The ANOVA analysis of the DeCyder software was used to identify statistically significant (p < 0.01) differences in protein abundance between the control and CP4 experimental groups using four biological repeats. A threshold value of at least a 1.2-fold difference in protein abundance was used.

## Results

### Effect of Plasma Treatment on Sunflower Seedling and Mature Plant Morphology

**Figure 1:**
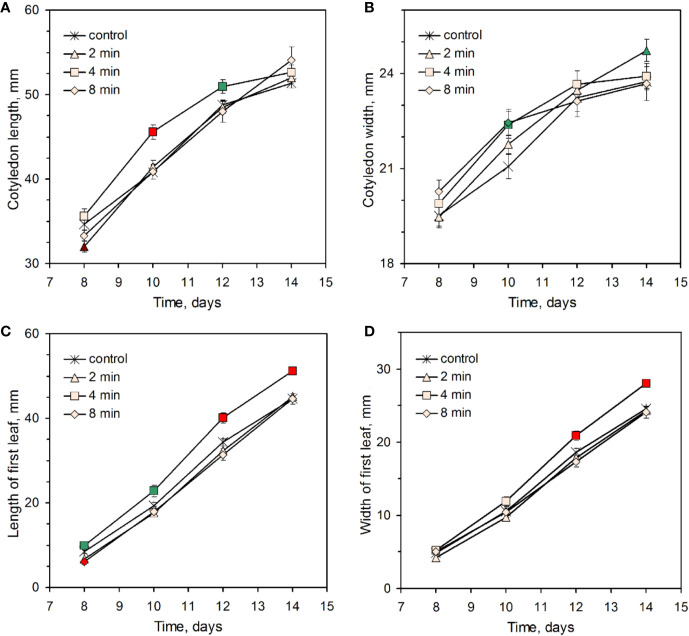
Effect of plasma treatment on sunflower seedling cotyledon **(A, B)** and first leaf **(C, D)** length **(A, C)**, and width **(B, D)**. Data from six independent experiments is presented as mean and standard error of the mean. Color of the symbols represent significant differences of the mean values compared to the control (green – p < 0.05, red – p < 0.01).

To establish an optimum CP treatment duration, sunflower seeds were placed at 3 mm distance from the DBD plasma source and irradiated for 2, 4, or 8 min cumulative duration. For the morphology analysis, 150–220 seedlings from 6 independent experiments were used for each experimental group. Meanwhile, during the 2 weeks of seedling growth, the height of the seedlings remained similar in all experimental groups (**Supporting material Figures S1** and **S2**), the effect of CP treatment emerged as a difference in size of cotyledons and first true leaf (**Figure 1**). The most significant difference in length of cotyledons was reached at day 10 for the CP 4 min group which had an 11.6% **±** 2.1% higher mean value compared to the control. This difference was lost when the cotyledons reached their maximum length after 2 weeks. On the contrary, the first true leaf dimensions of the CP4 group remained consistently larger during the experiment. At the end of the experiment, the mean value of leaf length and width was 14.6% **±** 2.1% and 14.3% ± 2.0% larger compared to the control, respectively. It is notable that shorter (2 min) or longer (8 min) treatment duration resulted in a complete loss of the growth-promoting effect, but without a significant adverse effect on plant morphology. Very similar length of hypocotyls for control and treated experimental groups suggests that germination timing was not affected by CP treatment.

Further, the long-term effect of CP-induced leaf growth stimulation was investigated in field experiments with plants grown to maturity. The size of the five true leaves was measured after 2 months of cultivation. Taking into consideration leaf size variations depending on age, a relative value was assessed for length and width of each consecutive leaf as compared to the control, and the derived cumulative values are shown in **Table 1**. In agreement with the results obtained in the experiments with sunflower seedlings, an average leaf length and width dimensions were significantly larger compared to the control (8.1% **±** 1.8% and 9.9% ± 2.5%, respectively) upon the CP 4 min treatment, and the stimulating effect was abolished by the longer treatment duration. Furthermore, plants of the CP 4 min group yielded 12.7% ± 4.1% larger capitulum. Meanwhile, the mean value of plant height remained within a margin of error among the three experimental groups.

**Table 1:** Effect of CP treatment on sunflower mature plant morphology.

Parameter	Treatment		
	Control	CP 4 min	CP 8 min
Relative leave length, % ^a^	100.0 **±** 1.4 (n=284)	**108.1 ± 1.8** * (n=164; p=0.0006)	100.4 ± 2.1 (n=142)
Relative leave width, % ^a^	100.0 **±** 1.7 (n=282)	**109.9 ± 2.5** * (n=164; p=0.001)	98.0 ± 2.4 (n=142)
Area of capitulum, cm ^b^	747.7 ± 26.4 (n=33)	**842.5 ± 30.4** * (n=37; p=0.02)	754.8 ± 34.3(n=35)
Plant height, cm	221.8 ± 3.9 (n=38)	218.0 ± 3.3 (n=37)	228.7 ± 6.7 (n=35)

^a^a cumulative value of relative dimensions compared to the control was estimated separately for five true leaves after 2 months. ^b^measured after 3 months. Data is presented as mean and standard error of the mean. Asterisk indicates mean values significantly different compared to the control (p < 0.05).

Since the extraction of roots of mature plants grown in field or expanded seedlings grown in peat substrate could have an adverse effect on the precision of root measurements, the effect of CP treatment on root development was assessed at an early stage of seedling development (4 days after seedling emergence) while the main root could be easily extracted intact (**Supporting material Figure S1**). Roots of the seedlings germinated from the CP 4 min treated seeds were 8.7% ± 2.7% (p=0.014) longer [6.5 ± 0.15 (n=133) and 7.1 ± 0.17 (n=143) cm for the control and treated seedlings, respectively]. In agreement with the experiments described above, no significant effect on hypocotyl length [1.9 ± 0.04 (n=136) and 1.9 ± 0.03 (n=146) cm for the control and treated group, respectively] was detected. Size of the cotyledons was not examined as it closely corresponded to seed size at this growth stage.

### CP Treatment-Induced Changes in Plant-Associated Bacteria Community Structure

To investigate bacterial metagenomic diversity, six DNA pools were extracted from the control and CP-treated roots and cotyledons of the emerging seedlings, and leaves of 2-week-old seedlings. The samples were subjected to 16S rRNA metagenomic analysis using Ion Torrent sequencing platform. Sequencing and read mapping statistics are presented in **Supporting material Table S1**. For the six samples, 5,165,808 raw reads were generated in total. Following demultiplexing, quality trimming and chimera removal of initial sequence reads, from 404,580 to 879,930 sequences were collected for each sample (3,509,611 in total) of high-quality sequence with a read length from 235 to 243 bp.

After rarefaction at a depth of 29,710 sequences per sample, a total of 487 distinct OTUs were obtained with 97% similarity cutoff for all samples. The detected OTUs were assigned into 13 phyla, 27 classes, 55 orders, 113 families, and 158 genera. At the phylum level, *Firmicutes* was dominant in all samples (**Supporting material Figure S3**), though it was more prevalent in CP-treated samples as compared to the control. In consecutive order of abundance, other phyla included *Proteobacteria*, *Actinobacteria* and *Bacteriodetes*, and these had a similar distribution among the samples. An exception was the class of *Gammaproteobacteria* which was observed only in root (control and treated) and control cotyledon samples.

At a family level, OTUs were allocated to 113 different taxa; however, depending on the sample, 71% to 83% of the OTUs were assigned to three dominant families of *Bacillaceae*, *Rhodobacteraceae* and *Moraxellaceae*, and within these families only a small fraction (less than 1%) of the OTUs were assigned to the genus level. On the contrary, 66%–86% of the OTUs allocated to the remaining 110 families were assigned to a lower taxonomic level. This indicates that the majority of the sequences assigned to the three dominant families had low similarity to the microbial database used for read mapping and could include a significant proportion of plastid or mitochondrial sequences that are absent in the database and would be erroneously assigned to the related bacterial families. Following this line of reasoning and considering that the three dominant families were similarly abundant among the samples, this would be of low interest to pursue; therefore only OTUs assigned at the genus level were used for further analysis of microbial diversity.

To compare the richness and diversity of bacterial species in individual samples, alpha diversity indexes of Simpson, Shannon, Chao1 estimator and the observed species were used (**Supporting material Table S2**). A number of the observed OTUs varied from 41 to 114 and the highest number of OTUs was observed in roots and leaves of the control plants, whereas the CP4 treated cotyledons had the lowest number of OTUs. Rarefaction curves demonstrated that OTU abundance was diverse among the different samples (**Supporting material Figure S4**), and the saturating number of OTUs indicated that bacterial communities were sufficiently sampled.

Based on the alpha diversity indexes (**Supporting material Table S2**), the control leaves and roots had similar microbial richness, which was the highest among all samples. The richness was reduced in leaf samples after CP treatment (approximately two-fold for the Chao1 and 4.5-fold for H’ and S) but only slightly affected in the roots. Meanwhile, the microbial richness of cotyledons was the lowest among the control samples, and it was further reduced after CP treatment but the effect was lower compared to leaf samples (approximately 1.5-fold for all the indexes).

**Figure 2:**
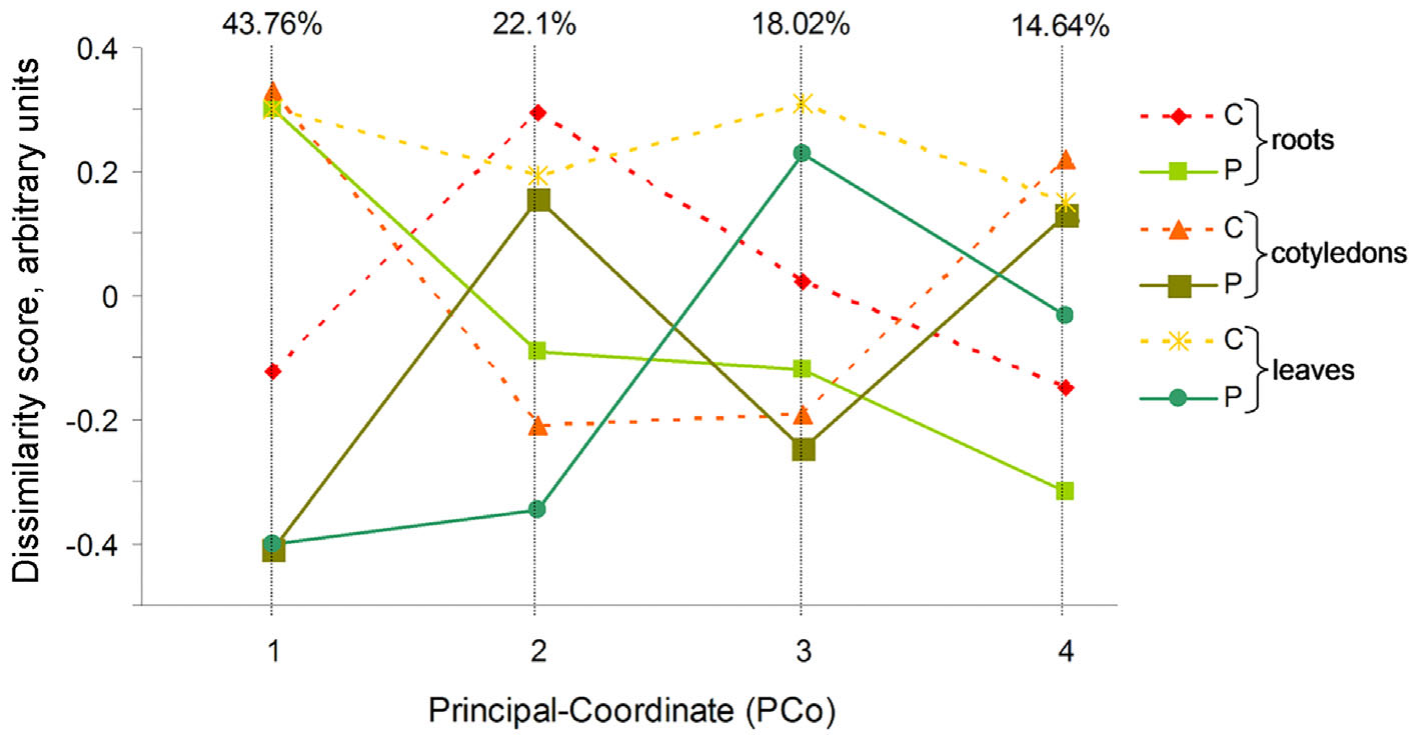
Variability within the data set of 16S rRNA metagenomic analysis projected in a set of four uncorrelated axes of the principal coordinate analysis (PCoA) carried out using Bray-Curtis dissimilarity index. Proportion of variance for each dimension is indicated on top. For each axis, objects ordinated closer to one another are more similar than those ordinated further away.

The Bray–Curtis dissimilarity matrix of beta diversity was calculated to differentiate the bacterial communities at the genus level among the control and CP-treated sunflower samples (**Figure 2**). The first four components comprised a larger than 98.5% variation. The first and second axes include the largest variability (65.86% in total) and mainly reflect differences between microbiome of sunflower seedlings germinated from the plasma-treated seeds (CP, solid line) and control seedlings (C, dashed line). For the remaining two axes that include 32.66% of variability, the control and CP-treated data points are projected more closely and the differences related to distinct tissues used in the analysis are more prominent. There was little association between the experimental groups representing different plant organs or the effect of CP treatment. Almost half (43.8%) of the detected variation was included in the first component representing a strong clustering of the control leaf and cotyledon groups, with the CP-treated root experimental group as well as CP-treated leaf and cotyledon groups at the extremes of the axis. The two extremes of the second component (22.1% variation) represented an association between control root and leaf with CP-treated cotyledon experimental groups and the control cotyledons and CP-treated root and leaf samples.

The differences detected by PCoA were illustrated by the Petal map based on distribution of the OTUs (**Supporting material Figure S5**). The analysis revealed that the control and CP treated samples of different parts of sunflower share or has distinct bacterial OTUs. The diagram revealed 29 core OTUs including *Mycobacterium*, *Rhodococcus*, *Nocardioides*, *Solimonas*, *Curtobacterium*, *Candidatus Portiera*, and *Pseudomonas* as the most common genera. Interestingly, the number of unique OTUs for the control leaves of sunflower contrastingly differed from those treated with CP and the number of OTUs in CP-treated cotyledons were also extremely low.

**Figure 3:**
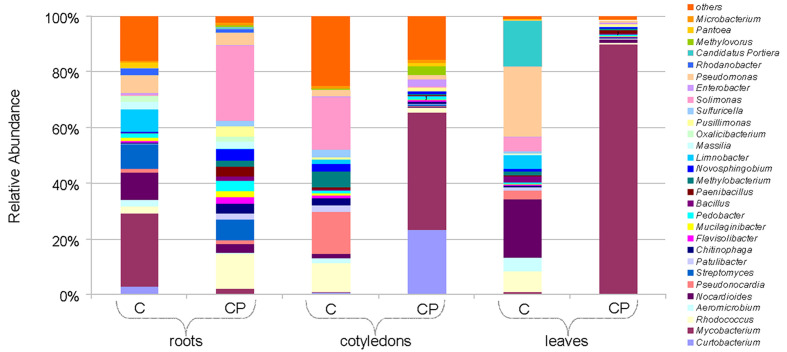
Bacterial composition at a genus-level for the *16S rRNA* gene in control (C) and cold plasma treated (CP) sunflower samples. “Others” group includes minor genus with <0.5% of total abundance.

Differences in bacterial abundance at the genus level are shown as a heatmap in **Supporting material Table S5**. Among the 158 bacterial genera identified across all samples, the distribution of the 29 most common (>0.5% of the total abundance) genera was summarized in **Figure 3**. Major variation was observed for the *Mycobacterium* genus that was greatly enriched in the leaf (28% of total sequences) and cotyledon (4.3%) samples upon CP treatment, meanwhile a considerable proportion (4%) of the sequences assigned to the genus in the control root samples was reduced approximately ten-fold after CP treatment. Other notable differences observed after CP treatment included a large relative increase in the abundance of *Solimonas* and *Rhodococcus* spp. in roots, *Curtobacterium* spp. in cotyledons, and a decrease in abundance of the dominant (>1% of total sequences) *Solimonas*, *Pseudonocardia*, and *Rhodococcus* spp. in cotyledons as well as a decrease in the abundance of *Pseudomonas*, *Nocardioides*, *Rhodococcus*, and *Candidatus Portiera* spp. in leaves.

### Differential Protein Expression in Sunflower Seedling Roots

**Figure 4:**
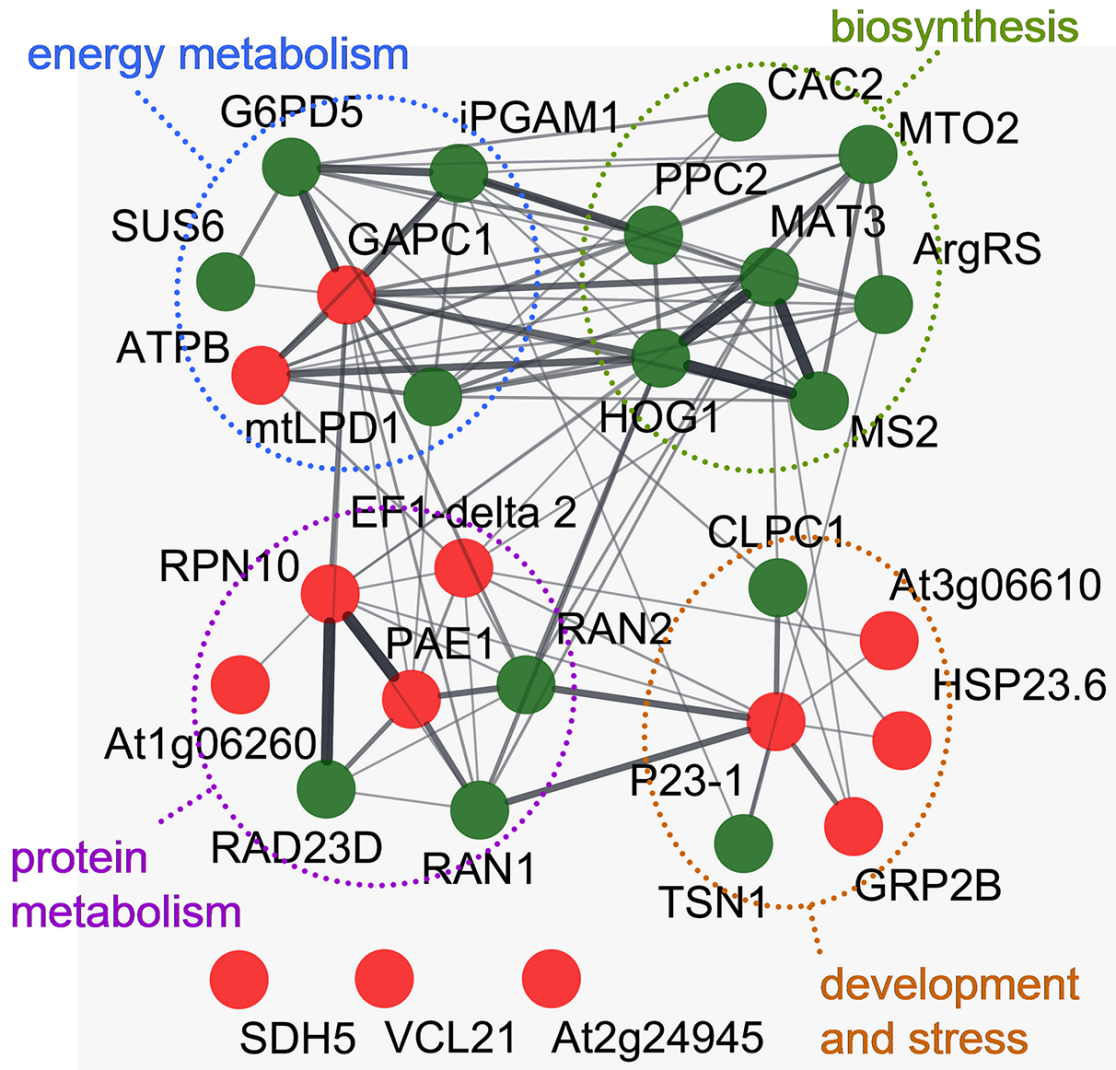
Interaction network of proteins differentially expressed in roots of sunflower seedlings germinated from the CP-treated and control seeds. The network was built using the String database using Arabidopsis homologues related to the identified sunflower proteins. Node base color represents a decrease (green) or increase (red) of protein abundance compared to the control. The thickness of the connecting lines represents significance of the interaction. Three nodes representing proteins that lack significant interactions within the network are shown at the bottom. Four distinct functional clusters are enclosed by colored dashed lines.

Proteomic analysis was used to assess differential protein expression in roots of sunflower seedlings germinated from the seeds treated with CP for 4 min. An acidic range of pH 4–7, corresponding to dominant pI value of cytosolic proteins (Schwartz et al., 2001; Wu et al., 2006), was selected for fractionation using isoelectrical focusing. After gel alignment, the average number of detected protein spots was 2351 ± 314 per gel (**Supporting material Figure S6**). Among the 67 differentially expressed proteoforms, more than one-third (26) were significantly upregulated by CP treatment and 41 were downregulated in the roots of the emerging seedlings (**Supporting material Figure S7**). Peptide fingerprinting of trypsin digested proteins identified 29 unique proteins (**Supporting material Tables S3** and **S4**). Analysis of interactions among the identified proteins assessed using Arabidopsis homologues and the String database revealed a network of 26 proteins that was partitioned into four functional clusters mostly linked to biosynthesis of methionine, threonine, or amino acids derived compounds, lipid biosynthesis and protein metabolisms including stress response related proteins as well as carbon fixation and energy metabolism (**Figure 4**). No significant interactions were identified for vicilin-like seed storage protein (VCL21) and two other proteins with undefined biological function; however it is possible that as a member of 7S globulins family that contains salycilic acid binding and putative superoxide dismutase structural motifs VCL21could have a role in the regulation of oxidative stress response (Shikhi et al., 2018) and the enzyme succinate dehydrogenase 5 (SDH5) is related to energy metabolism in mitochondria.

## Discussion

Our study established that CP irradiation of common sunflower seeds stimulates root and lateral organ growth. Plant leaves or flowers originate from primordia cells at the peripheral zone of the shoot apical meristem, and their formation proceeds by gradual cell proliferation and expansion (Granier and Tardieu, 1998). The lateral organ growth is controlled by a network of developmental genes, miRNA and phytohormones [reviewed by Maugarny-Cales and Laufs, (2018)], and their final size is modulated by environmental factors, balance between water uptake in roots and transpiration in leaves, and direct signals from the roots (Bogre et al., 2008; Chitwood and Sinha, 2016). The water supply from roots also modulates stomatal development and opening (Pinheiro and Chaves, 2010; Hepworth et al., 2016) which by itself is the main regulator of gas exchange in leaves. Interestingly, an increase in the photosynthetic efficiency in sunflower leaves induced by seed treatment with CP has been recently documented (Zukiene et al., 2019), and it is likely a consequence of the improved gas exchange due to enhanced root growth and supply of water. Therefore it could be proposed that the CP-induced lateral organ growth stimulation in sunflower is likely the result of improved water supply due to enhanced root growth, and the detected modulation of energy metabolism, biosynthesis, developmental and stress response processes that leads to root growth stimulation is linked to the changes in the microbial assembly of the roots observed after CP-treatment.

Plant-associated bacteria play an important role in seed germination and plant growth, by supplying nutrients or priming systemic disease resistance (Mendes et al., 2013; Tamosiune et al., 2019). Microbiota has profound effect on plant and agro-ecosystem health and the reduced microbial diversity is often linked to spread of pathogenic microorganisms (Sessitsch and Mitter, 2015; Berg et al., 2017). Our recent study demonstrated that that CP treatment of seeds results in changes of composition of *Arabidopsis thaliana* seedling and mature plant microbiota that might be of importance for plant growth regulation (Tamosiune et al., 2020). In the current study, metagenomic analysis demonstrated that CP treatment results in reduction of plant-associated microbial diversity in sunflower seedlings and had been mostly represented by high abundance of *Mycobacterium* spp. in cotyledons and subsequent expansion of the species to a single dominant taxa in the leaves of 2 weeks old seedlings (**Figure 2**). This phenomenon had no apparent adverse effects on health of the seedlings. Although microbial diversity was not assessed in the field grown plants, their growth pattern and response to CP treatment was similar to the seedlings grown under controlled conditions, suggesting that disease suppression might not be related to relatively isolated laboratory environment. Possibly, the composition of microbial community essential to maintain a normal health status was preserved in the treated plants or/and compensated by large microbial diversity in roots where CP-induced changes in microbial composition but the level of overall diversity was maintained similar to control seedlings. However, the provided evidence of CP-induced changes in plant microbial diversity and its composition raises necessity to address long-term impact of the new CP-based plant growth-enhancing technologies on the plant and soil environment microbial diversity in further studies.

The actinobacteria *Mycobacterium* genus is rather common in the rhizosphere and plant tissues (Conn and Franco, 2004; Pirttila et al., 2005; Mano et al., 2007; Quambusch et al., 2014; Wang et al., 2016; Bouam et al., 2018) but its occurrence in plants has been underestimated due to the requirement for special media growth conditions (Mano et al., 2007). A diversity of the *Mycobacterium* species has been documented for rice and tomato plants, as well as *in vitro* cultures (Koskimaki et al., 2010; Wang et al., 2016; Bouam et al., 2018). Some species of *Mycobacterium* have been reported to suppress undifferentiated Scots pine tissue growth *in vitro* and reduce the length of hypocotyls of seedlings, but similar negative effects have not been observed for other species (Laukkanen et al., 2000). Proliferation or metabolic activity of the bacterium has been linked to the development of Scots pine buds (Pirttila et al., 2005). Considering the above, it could be presumed that, in our study, the *Mycobacterium* genus might be represented by several distinct species, some of which could possibly be linked to the observed growth modulating activity.

It is also notable that *Mycobacterium* spp. have not been detected in the seeds of sunflower (Leff et al., 2017) or rice (Wang et al., 2016), suggesting that the bacterium or its spores can only populate the seed surface and/or it colonizes plants from the soil environment. Antimicrobial effect of CP has been well established [see review by Patil et al., (2016)], however, recent findings by Ji et al., (2019) have shown that CP treatment could stimulate the activity of plant growth-promoting bacteria as well. Therefore, depending on the dose and the composition of CP-generated reactive species, CP treatment could trigger a multifaceted response of seed microflora. Thus it could be presumed that the resistance of spore-forming *Mycobacterium* spp. to CP treatment could benefit survival on the seed surface and subsequent proliferation of the bacterium. On the contrary, the abundance of non spore-forming *Rodococcus*, *Nocardioides*, *Pseudomonas*, and *Candidatus Portiera* spp., which dominate leaf bacterial assembly of the control plants, has been largely reduced by CP-treatment. An interesting exception might be the genus *Candidatus Portiera* which has been described as an endophyte in the Citrus greening disease pathogen infected plants (Sagaram et al., 2009). The bacterium is highly adapted to endosymbionic life in bacteriocytes of whiteflies and entail strict vertical transmission from mother to offspring (Baumann, 2005), therefore its transmission through seeds and proliferation within the plant is unlikely. Higher abundance of the bacterium in the leaves of the control plants might suggest reduced incidence of the bacterium infiltration by the insect vector or reduced contamination of samples with whitefly eggs upon CP treatment in our experiments.

The resistance of spores to CP treatment and their role in bacterial transfer from seeds to plants does not explain the reduced occurrence of spore-forming *Mycobacterium* spp. and increased abundance of non spore-forming *Solimonas* and *Rodococcus* spp. in CP-treated root samples. It appears that root colonization from the rhizosphere prevails over the seed-originated bacterial community during an extended period of plant growth. However, it appears that the effect of CP treatment plays a significant role in shaping the composition of the root microbiome and the associated biological interactions within the microbial community, or with a plant-host which might account for the observed plant growth stimulating effect.

The most prominent CP-induced quantitative difference in sunflower root bacterial community composition is reflected by a shift in balance between the occurrence of actinobacteria *Mycobacterium* and *Rodococcus* spp., as well as a reduced incidence of *Limnobacter* spp., and an increase in the abundance of *Solimonas* spp. The latter bacterium has been initially isolated from soil (Kim et al., 2014) but is also abundant in freshwater (Zhou et al., 2014). *Solimonas* spp. biological interactions in the plant rhizosphere have not been documented but the ability to decompose complex organic compounds, including chemical pollutants (Zhou et al., 2014), might be of importance. The rhizosphere is populated with microorganisms that survive on compounds released from plant roots and are able to actively regulate the production of plant root exudates (Phillips et al., 2004). The export of exudates from the roots is also critical to maintain balance of pathogenic and beneficial microorganisms in the rhizosphere (Hennion et al., 2019). The detected differential expression of proteins involved in amino acid, lipid, sugar biosynthesis and protein turnover processes, as well as the development and stress response in sunflower seedling roots, could be related to CP-induced changes in the microbial community of the rhizosphere.

The overall occurrence and abundance of bacterial taxa detected in sunflower plants was significantly different compared to the previously described composition of sunflower microbiome (Leff et al., 2017). This might arise from the differences in genotype and the growing conditions used, and it could be anticipated that the outcome of CP treatment might depend on these circumstances as well. In addition, biodegradation or biosynthesis of organic compounds is enhanced by bacterial and fungal interactions (Jayasinghearachchi and Seneviratne, 2004; Seneviratne et al., 2006), and bacteria have also been shown to promote the growth of mycorrhizal fungi (Frey-Klett et al., 2007). Similar interactions in the sunflower rhizosphere might benefit formation of arbuscular mycorrhiza with *Rhizoglomus irregulare* or other fungi (Leff et al., 2017). The fungal community remains unaccounted for in our study; therefore further microbiome analysis could provide additional insights into the complex biological interactions modulated by CP treatment of seeds.

Protein abundance differences induced by CP treatment were consistent but had a characteristically low amplitude which had been observed in studies on low intensity stress (eustress) stimuli such as low intensity UV-B treatment (Aksoy et al., 2010), or low-pressure capacitively coupled plasma or electromagnetic field treatment of sunflower seeds (Mildaziene et al., 2019). However, the protein expression pattern induced by DBD type plasma source was very different from the previous results obtained using low-pressure capacitively coupled plasma (Mildaziene et al., 2019). While the latter method had no significant effect on root protein abundance (Mildaziene et al., 2019), in the current study, extensive protein expression variation was detected in seedling roots upon DBD type CP treatment. The difference might arise from the presence of atmospheric pressure air in the DBD type CP that is a source of abundant reactive species generated during seed treatment.

While the mechanism of action of CP-generated reactive species on plants is poorly understood, it is presumed that plant response to seed treatment is a consequence of the stressor-induced eustress phenomena. ROS stressor-induced plant growth promoting effect for wheat plants germinated from H_2_O_2_ treated seeds had been described previously (Abdel Latef et al., 2019). It is notable that the H_2_O_2_ treatment stimulated shoot elongation (Abdel Latef et al., 2019) which was not observed in our study, suggesting a different mechanism of growth stimulation by CP treatment. Meanwhile the physical stimuli could have direct effect on seed germination, the lateral organ growth stimulation is likely linked to the long term changes in the composition of microbial population as a consequence of the antimicrobial activity of reactive species, such as O_3_ or·OH, produced by atmospheric pressure type plasma source. Furthermore, the growth promoting effect has been abolished by longer treatment duration without an adverse effect on plant growth, suggesting these effects could be dependent on fine modulation of concentration and/or composition oft CP-produced reactive species.

**Figure 5:**
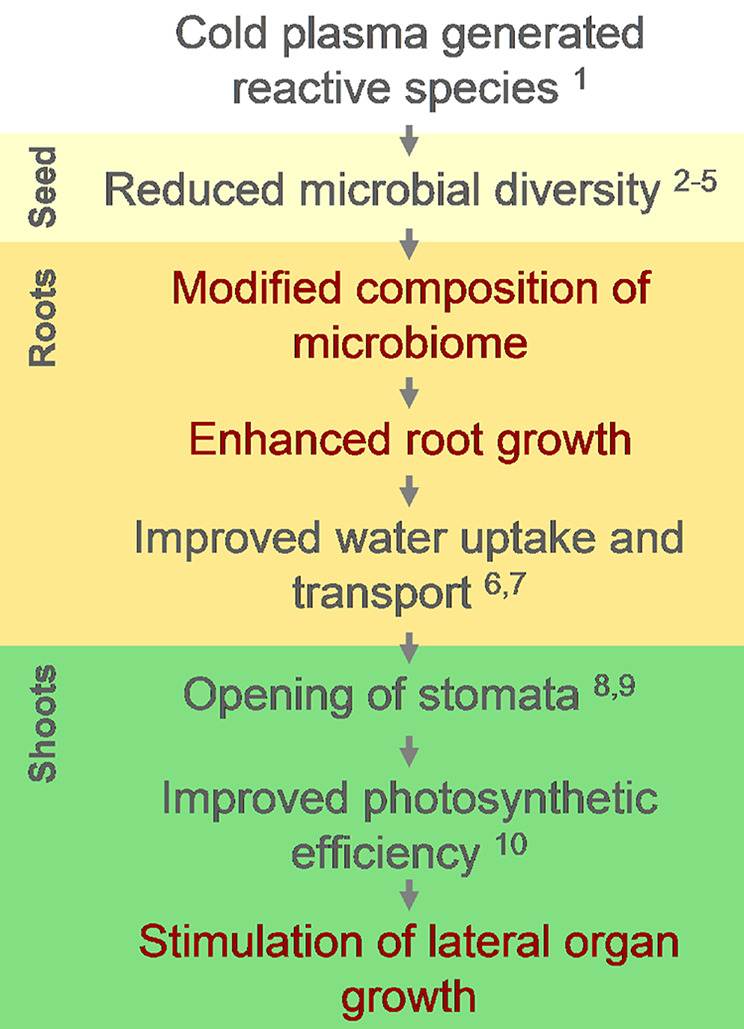
A diagram representing the sequence of events which leads from impact of CP-generated reactive species on seed microbiome to stimulation of lateral organ growth in common sunflower and is derived from results observed in current (red font) or previously published studies (black) indicated by numbers as follow: 1—(Whitehead, 2016); 2—(Patil et al., 2016); 3—(Han et al., 2016); 4—(Hong et al., 2009); 5—(Reineke et al., 2015); 6—(Bogre et al., 2008); 7—(Chitwood and Sinha, 2016); 8—(Pinheiro and Chaves, 2010); 9—(Hepworth et al., 2016); 10—(Zukiene et al., 2019).

Taken together, our study has established that, under optimum treatment conditions, the atmospheric pressure DBD type CP irradiation of sunflower seeds stimulated growth of seedling roots and above ground lateral organs. The metagenomic analysis of the seedling microbiome revealed changes in the structure of plant-associated bacteria population after CP treatment. For the above ground tissues, the consequences of the treatment appears to be related to inactivation of non-spore forming bacteria on seeds by the CP-generated reactive species that leads to domination of spore forming genera, such as *Mycobacterium* spp., in the microbiomes of cotyledons and leaves of germinated seedlings. In the roots, the antimicrobial CP treatment effect is largely presided by the subsequent bacterial colonization from the soil but appears to have a long-term effect on microbial composition in the rhizosphere and/or endosphere which could be linked to stimulation of root elongation. Furthermore, growth stimulation of the roots is likely the basis for enhanced lateral organ growth due to an increase in water uptake and/or direct root signaling (**Figure 5**). Proteome analysis revealed consistent but low amplitude differences in protein abundance that imply a subtle modulation of root metabolic and stress response processes as a consequence of the changes in plant-associated microbiome upon CP treatment. Further examination of the interactions of soil microbial community could provide useful insights in the complex biological interactions in the rhizosphere that mediate the CP-induced long-term effect on plant phenotype.

## Data Availability Statement

The datasets presented in this study can be found in online repository of the NCBI Sequence Read Archive: https://www.ncbi.nlm.nih.gov/bioproject/PRJNA627824

## Author Contributions

IT, VM, KK, MS, and DB: conceptualized and designed the experiments. IT, DG, PH, VM, and DB: acquired, analyzed, and interpreted the data. IT and DB: drafted the manuscript. VM, KK, and MS: contributed to critical revision of the manuscript. All authors contributed to the article and approved the submitted version.

## Conflict of Interest

The authors declare that the research was conducted in the absence of any commercial or financial relationships that could be construed as a potential conflict of interest.

## Abbreviations

CP, cold plasma or high-frequency atmospheric-pressure plasma; CP2, CP4, CP8, CP irradiation of 2, 4, or 8 min, respectively; CTAB, cetyltrimethylammonium bromide; DBD, dielectric barrier discharge; H_2_O, water; H_2_O_2_, hydrogen peroxide; N_2_, molecular nitrogen; NGS, next-generation sequencing; NO_x_, nitrogen oxides; O_2_, molecular oxygen; ^-·^O_2_, superoxide anion; OH**^·^**, hydroxyl radical; ONOO^–^, peroxynitrite anion; OUT, operational taxonomic units; PcoA, principal coordinate analysis; ROS/RNS, reactive oxygen and nitrogen species; SDH5, succinate dehydrogenase 5; SDS, sodium dodecyl sulfate; VCL21, vicilin-like seed storage protein.
