# Supplementary material for: Cold Plasma Treatment of Sunflower Seeds Modulates Plant-Associated Microbiome and Stimulates Root and Lateral Organ Growth
Source: Front Plant Sci. 2020 Aug 28;11:568924. doi: 10.3389/fpls.2020.568924 (PMC7485318; doi:10.3389/fpls.2020.568924)
Supplement: Supplementary file 1 [file DataSheet_1.pdf]

Cold plasma treatment of sunflower seeds modulates plant-associated microbiome and stimulates root and lateral organ growth

Inga Tamošiūnė[1], Dalia Gelvonauskienė[1], Perttu Haimi[1], Vida Mildažienė[2], Kazunori Koga[3,4], Masaharu Shiratani[3], Danas Baniulis[1,*]

[1] Institute of Horticulture, Lithuanian Research Centre for Agriculture and Forestry, Babtai, Kaunas reg., Lithuania; [2] Faculty of Natural Sciences, Vytautas Magnus University, Kaunas, Lithuania; [3] Faculty of Information Science and Electrical Engineering, Kyushu University, Fukuoka, Japan; [4] Center for Novel Science Initiatives, National Institutes of Natural Sciences, Tokyo, Japan

[*] **Correspondence:** danas.baniulis@lammc.lt

Supporting information

Figures

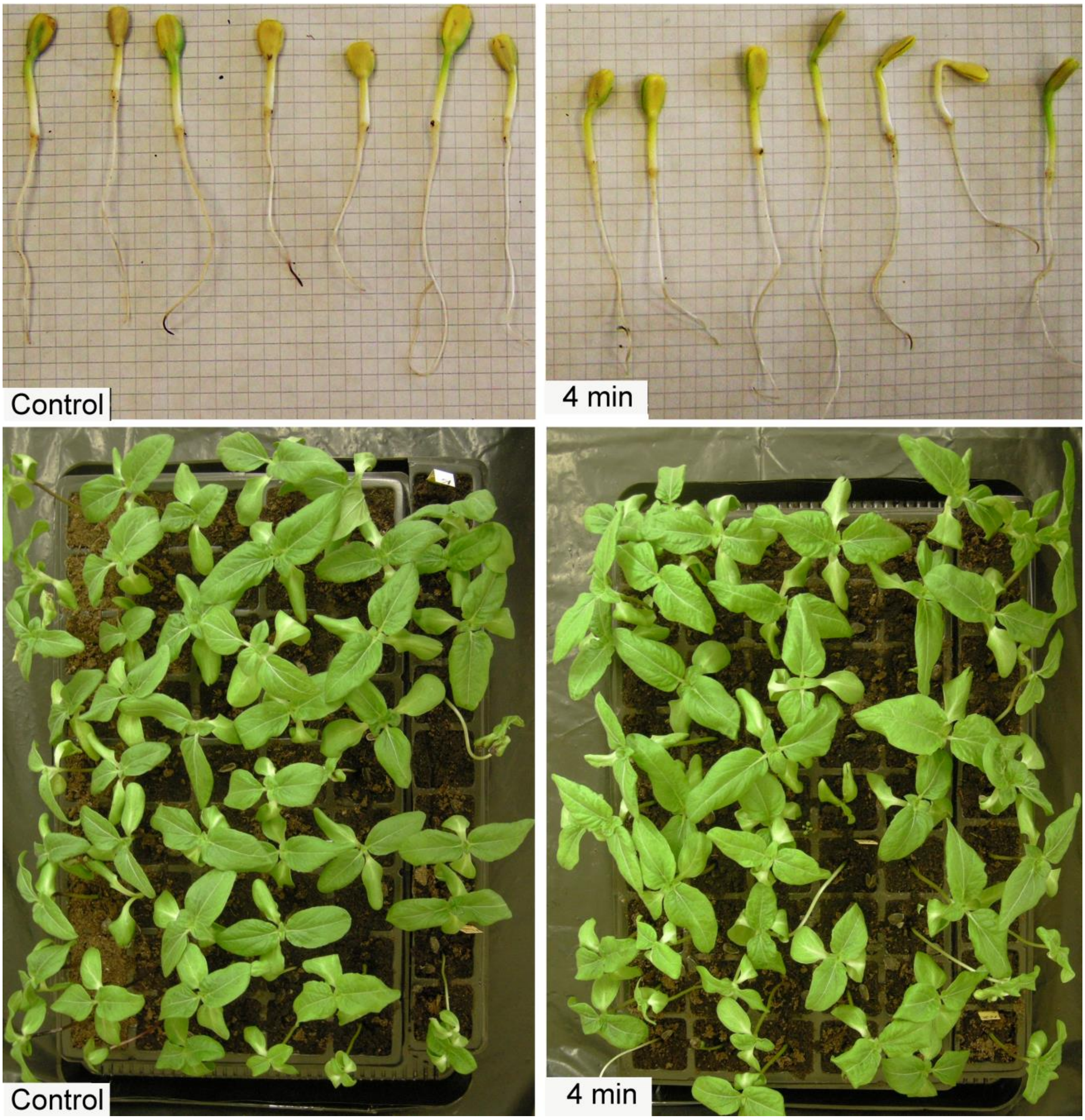

Figure S1. Representative samples of control (left) and 4 min CP treated (right) sunflower seedlings after 4 days (top) and 2 weeks of growth (bottom). For analysis of the emerging and 2 weeks old seedlings approx. 140 seedlings from 3 independent experiments 150-220 seedlings from 6 independent experiments were used for each experimental group, respectively.

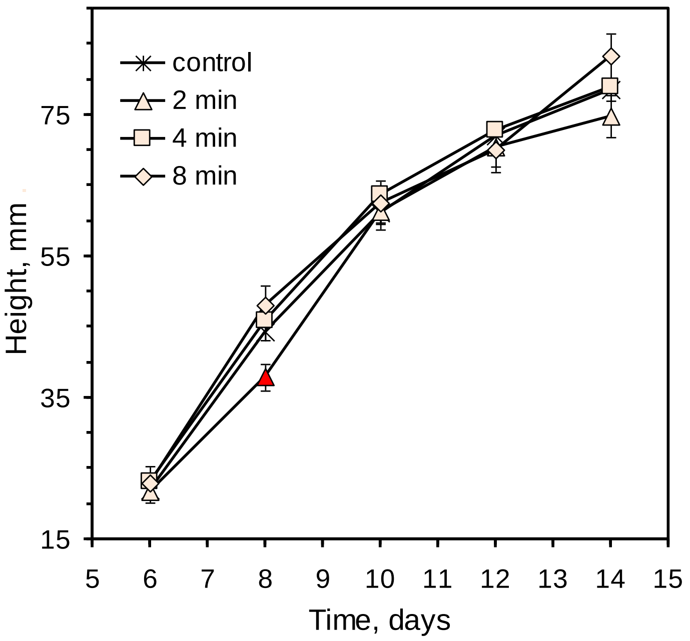

Figure S2. Effect of plasma treatment on sunflower seedling height. Data from 6 independent experiments is presented as mean and standard error of the mean. Red color of the symbol represent significantly ($p<0.01$) different mean value compared to control.

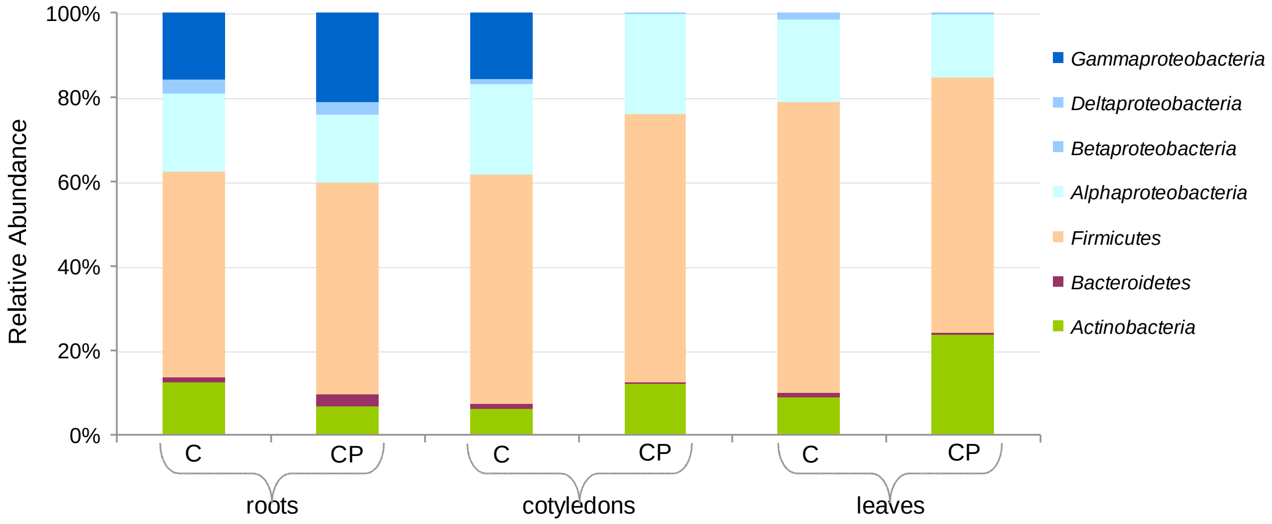

Figure S3. Bacterial diversity at phylum level of control (C) and sunflower seedlings germinated from cold plasma treated seeds (CP). *Proteobacteria* phylum is represented by classes of *alpha-*, *beta-*, *delta-* and *gamma-proteobacteria*.

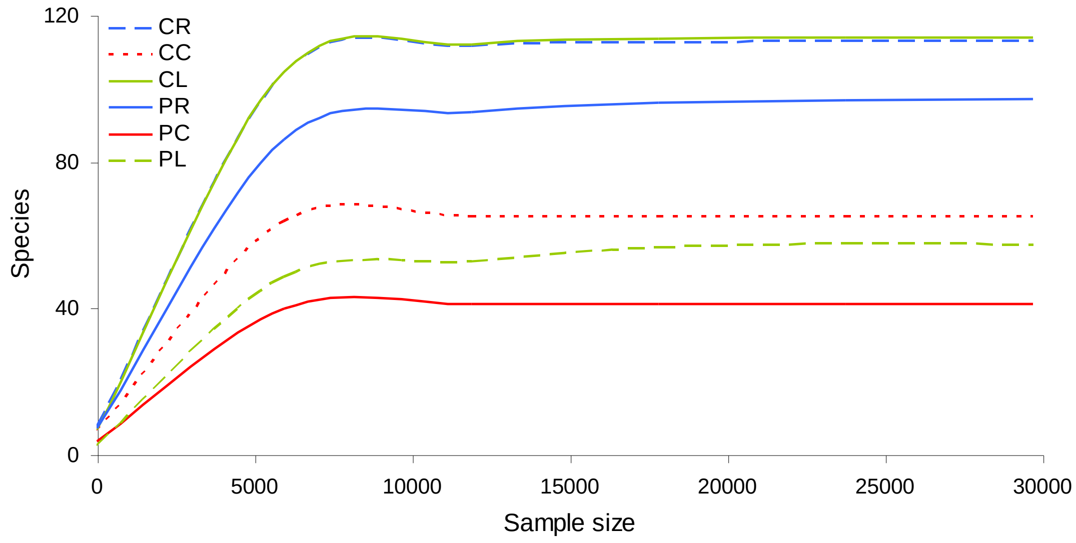

Figure S4. Rarefaction curves depicting the effect of 3% dissimilarity on the number of OTUs identified in control and sunflower seedlings germinated from cold plasma treated seeds. Abbreviations: CR, CC, CL – roots cotyledons and leaves of control, and PR, PC, PL – roots cotyledons and leaves of seedlings germinated from plasma treated seeds, respectively.

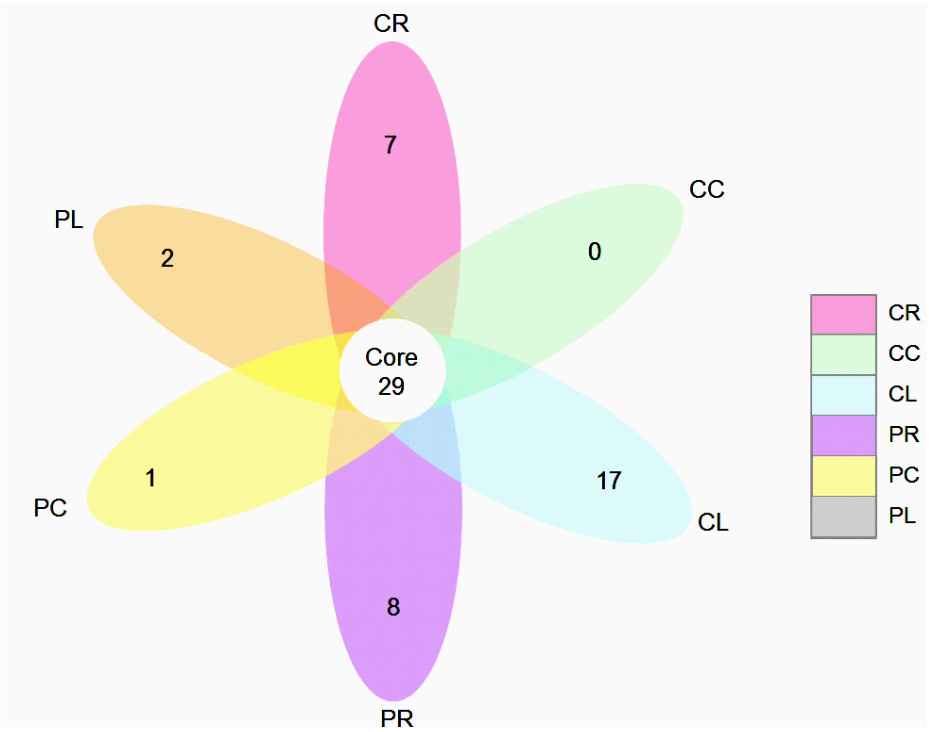

Figure S5. Distribution of Operational Taxonomic Units (OTUs) represented as petal map. The number on each petal represents the number of OTUs unique to the sample, and the middle core number represents the common OTUs to all samples. Abbreviations: CR, CC, CL – roots cotyledons and leaves of control, and PR, PC, PL – roots cotyledons and leaves of seedlings germinated from plasma treated seeds, respectively.

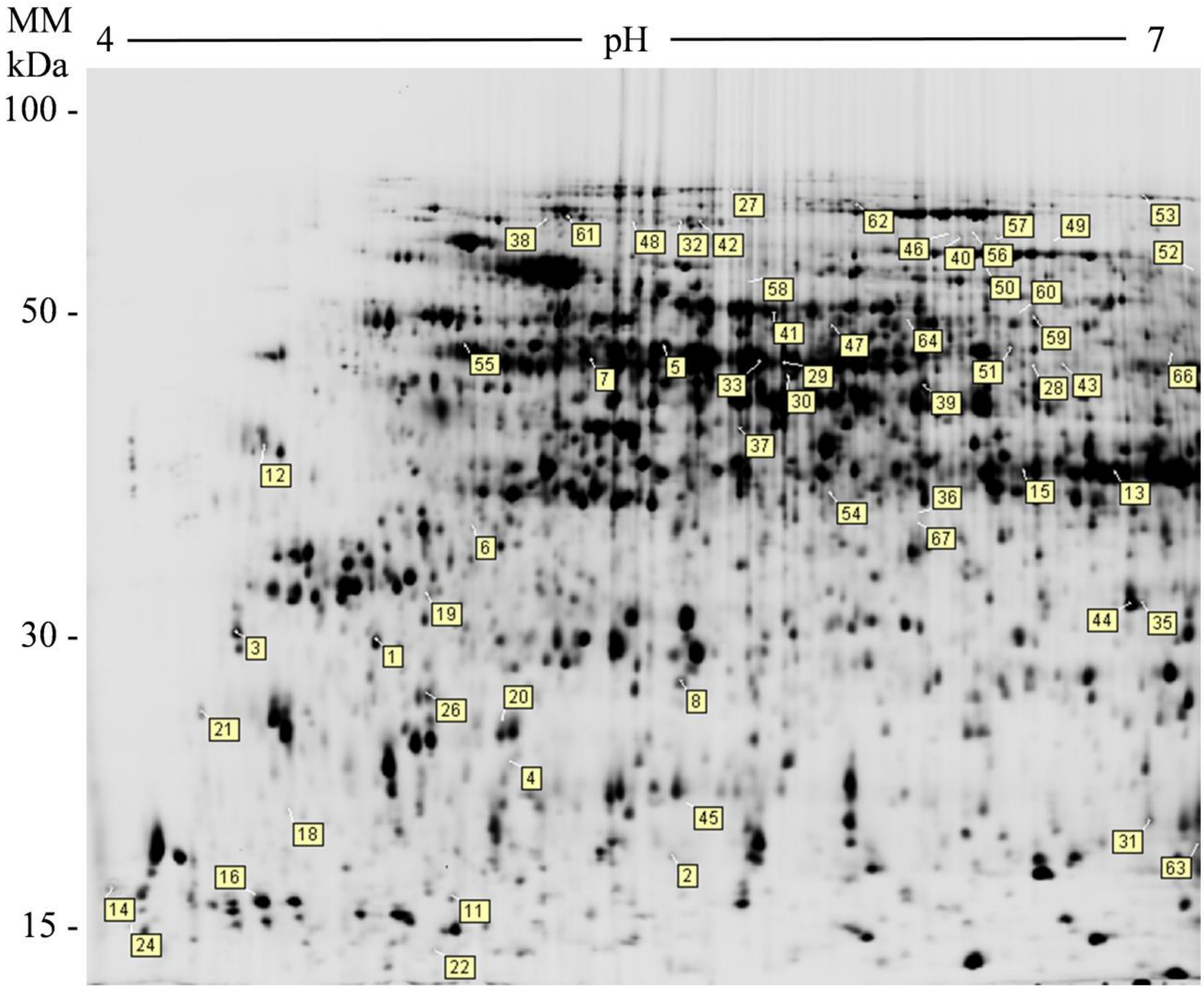

Figure S6. Representative results of the sunflower seedling root proteome analysis visualized as fluorescence of Cy2 dye labeled pooled sample of total cell protein. Protein sample (50 μg) was separated on the acidic pH 4–7 IPG strip and 10-16 % PAGE gel. Range of pH is indicated at the top, and the tentative molecular weight (MW) estimated based on theoretical MW of identified proteins is indicated on the left. Spots differentially expressed upon CP treatment are annotated with numbers.

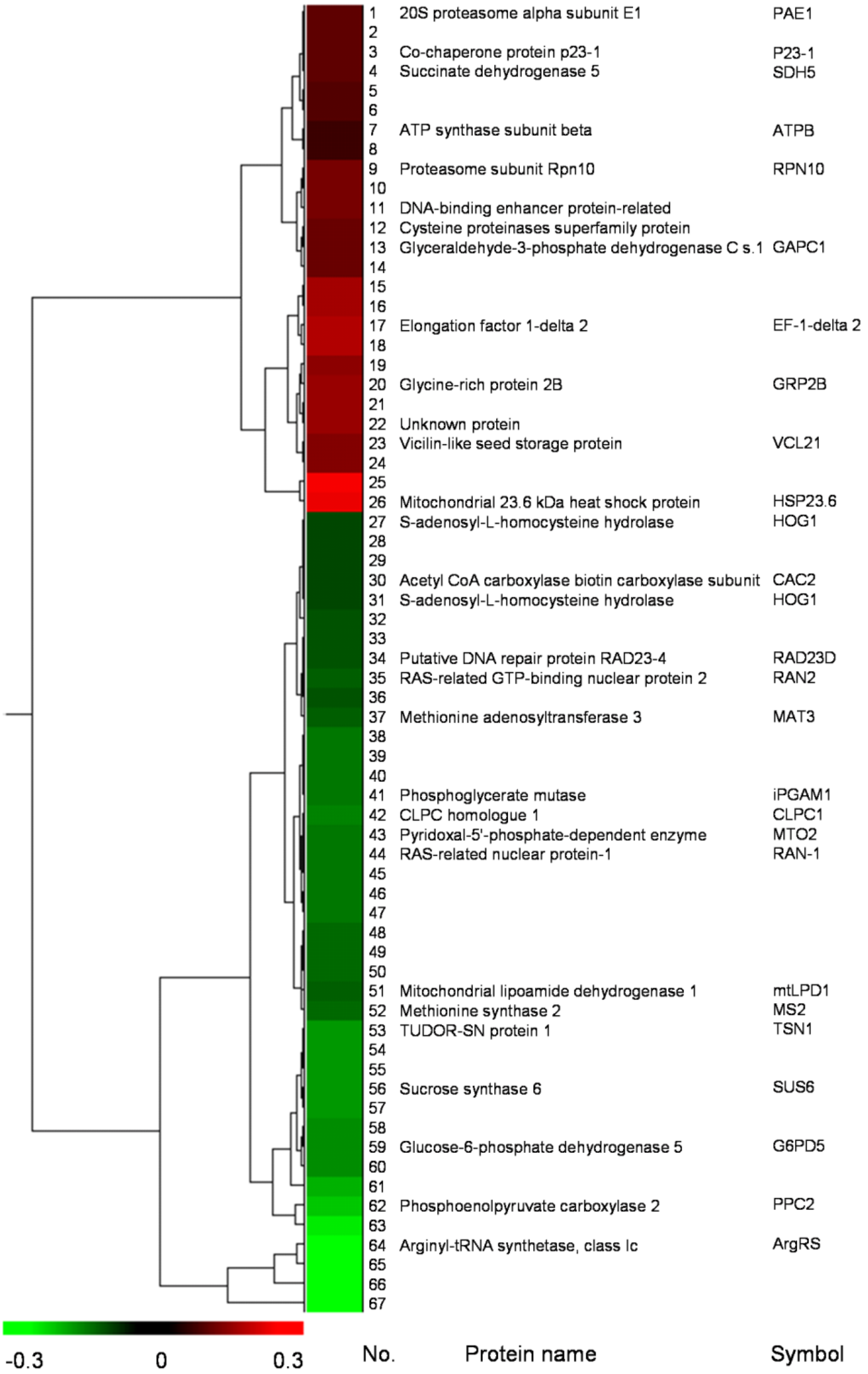

Figure S7. Hierarchical cluster analysis results of abundance data of the 67 proteoforms differentially expressed in the roots of emerging sunflower seedlings germinated from CP treated seeds. Colors indicate a decrease (green) or increase (red) in protein abundance compared to control. Spot number, protein name and symbol are shown in columns on the right.

Tables

Table S1. Summary statistics 16S rRNA metagenomic analysis

Parameter	Leaves		Cotyledons		Roots	
	Control	CP4	Control	CP4	Control	CP4
Mean read length	243	239	240	235	241	239
Total number of reads	800,426	1,317,067	562,131	637,157	828,258	1,020,769
Number of valid reads	566,477	879,930	404,580	434,445	545,034	679,145
Mapped reads in sample	423,470	713,037	325,906	345,552	388,863	508,152

Table S2. Microbiota alpha diversity estimates

Parameter	Leaves		Cotyledons		Roots	
	Control	CP4	Control	CP4	Control	CP4
Simpson index (S)	0.893	0.193	0.885	0.643	0.897	0.905
Shannon index (H’)	4.244	0.955	3.975	2.295	4.402	4.428
Chao1 estimator	114	58	65	41	113	97
Observed OTUs	114	57	65	41	113	97

Table S3. Results of identification and annotation of proteoforms differentially expressed in roots of emerging sunflower seedlings germinated from the CP-treated seeds

Proteo-form No.	Peptide ID at *H. annuus* genome database	Mascot score	Num. of pep-tides	SC [%]	MW / pI	Protein ID[1]	TAIR accession ID	Protein name	Protein symbol	Relative abundance
1	Ha412v1r1_15g048670	816	14	69,2	25.9/4.8	NP_001077717/ Q56ZL5	AT1G53850.1	20S proteasome alpha subunit E1	PAE1	1.28
3	Ha412v1r1_15g003650	265	7	40,8	24.7/4.5	NP_001154201/ Q8L7U4	AT4G02450.2	Co-chaperone protein p23-1	P23-1	1.28
4	Ha412v1r1_04g011770	182	5	32,9	25.8/7.8	NP_564506/ Q9SX77	AT1G47420.1	Succinate dehydrogenase 5	SDH5	1.29
7	Ha412v1r1_12g036700	3124	34	71,6	59.7/6.2	NP_051066/ P19366	ATCG00480.1	ATP synthase subunit beta	ATPB	1.21
9	Ha412v1r1_08g017240	496	9	36,5	41.8/4.5	NP_195575/ Q8L9G3	AT4G38630.1	Proteasome subunit Rpn10	RPN10	1.35
11	Ha412v1r1_11g050970	164	5	50,9	12.6/5.0	NP_566288/ Q9C901	AT3G06610.1	DNA-binding enhancer protein-related		1.36
12	Ha412v1r1_16g010050	901	11	26,2	50.5/5.1	NP_563764/ Q9LNC1	AT1G06260.1	Cysteine proteinases superfamily protein		1.31
13	Ha412v1r1_12g027260	1095	20	66,5	36.8/6.7	NP_187062/ P25858	AT3G04120.1	Glyceraldehyde-3-phosphate dehydrogenase C subunit 1	GAPC1	1.31
17	Ha412v1r1_09g053020	339	8	42,9	29.8/4.6	NP_179402/ Q7GA96	AT2G18110.1	Elongation factor 1-delta 2	EF-1-delta 2	1.53

20	Ha412v1r1_15g039850	103	3	29,3	19.3/5.7	NP_179702/ Q38896	AT2G21060.1	Glycine-rich protein 2B	GRP2B	1.43
22	Ha412v1r1_17g004340	224	4	56	10.8/5.1	NP_850060/ Q3EBU3	AT2G24945.1	Unknown protein		1.44
23	Ha412v1r1_14g002420	104	1	4,4	33.1/4.8	NP_179444/ Q9ZU69	AT2G18540.1	Vicilin-like seed storage protein	VCL21	1.39
26	Ha412v1r1_03g043170	525	9	40,4	24.7/5.5	NP_194250/ O23640	AT4G25200.1	23.6 kDa heat shock protein, mitochondrial	HSP23.6	1.70
27	Ha412v1r1_01g034440	670	16	46,6	53.1/5.5	NP_193130/ O23255	AT4G13940.1	S-adenosyl-L-homocysteine hydrolase	HOG1	-1.24
30	Ha412v1r1_04g023960	1611	27	69,5	57.4/6.2	NP_198386/ O04983	AT5G35360.3	Acetyl CoA carboxylase biotin carboxylase subunit	CAC2	-1.23
33	Ha412v1r1_01g034440	2315	36	77,7	53.1/5.5	NP_193130/ O23255	AT4G13940.1	S-adenosyl-L-homocysteine hydrolase	HOG1	-1.27
34	Ha412v1r1_15g017610	420	11	33,6	38.5/4.6	NP_198663/ Q84L30	AT5G38470.1	Putative DNA repair protein RAD23-4	RAD23D	-1.27
35	Ha412v1r1_01g046200	213	7	39,8	25.1/6.4	NP_197502/ P41917	AT5G20020.1	RAS-related GTP-binding nuclear protein 2	RAN2	-1.27
37	Ha412v1r1_14g044280	934	15	71,6	37.3/6.4	NP_181225/ Q9SJL8	AT2G36880.2	Methionine adenosyltransferase 3	MAT3	-1.28
41	Ha412v1r1_15g001200	725	16	46	60.5/5.5	NP_563852/ O04499	AT1G09780.1	Phosphoglycerate mutase	iPGAM1	-1.34
42	Ha412v1r1_08g042080	1472	32	48,5	98.7/5.8	NP_568746/ Q9FI56	AT5G50920.1	CLPC homologue 1	CLPC1	-1.37

43	Ha412v1r1_02g000320	103	3	8	55.5/6.9	NP_194713/ Q39144	AT4G29840.1	Pyridoxal-5'-phosphate-dependent enzyme family protein	MTO2	-1.36
44	Ha412v1r1_02g027140	586	12	70,1	25.2/6.4	NP_197501/ P41916	AT5G20010.1	RAS-related nuclear protein-1 |	RAN-1	-1.36
51	Ha412v1r1_07g006480	1064	22	63,6	52.6/6.7	NP_175237/ Q9LNF3	AT1G48030.2	Lipoamide dehydrogenase 1, mitochondrial	mtLPD1	-1.30
52	Ha412v1r1_07g025350	262	6	8,8	94.8/7.0	NP_187028/ Q94BN4	AT3G03780.3	Methionine synthase 2	MS2	-1.32
53	Ha412v1r1_09g054580	530	18	24,1	109.1/6.8	NP_196352/ Q8VZG7	AT5G07350.2	TUDOR-SN protein 1	TSN1	-1.44
56	Ha412v1r1_10g069800	508	15	24,9	93.2/6.0	NP_177480/ Q9FX32	AT1G73370.1	Sucrose synthase 6	SUS6	-1.46
59	Ha412v1r1_17g021970	695	17	68,9	59.1/6.2	NP_189366/ Q8VZD0	AT3G27300.2	Glucose-6-phosphate dehydrogenase 5	G6PD5	-1.4
62	Ha412v1r1_11g021050	1119	37	50,3	110.3/5.7	NP_850373/ Q5GM68	AT2G42600.1	Phosphoenolpyruvate carboxylase 2	PPC2	-1.60
64	Ha412v1r1_03g035210	193	4	9,3	66.5/6.0	NP_176826/ O23246	AT1G66530.1	Arginyl-tRNA synthetase, class Ic	ArgRS	-1.83

[1] NCBI RefSeq or UniProtKB/Swiss-Prot accession number provided at the NCBI Protein database. [2] Mean values of statistically significant differences in relative abundance compared to control. Abbreviations: SC – sequence coverage; MW – molecular weight; pI – isoelectric point.

Table S4. String database GO term enrichment analysis results (FDR < 0.01) for the 26 unique proteins differentially expressed in the roots of germinating seedlings

**Term name**	**Description**	**FDR value**	**Enriched genes**
GO:0046686	response to cadmium ion	1.73E-10	iPGAM1, mtLPD1, PAE1, At3g03780, GAPC1, G6PD5, HSP23.6, TSN1, RAN-1, RAN2
GO:0044281	small molecule metabolic process	2.33E-06	iPGAM1, SDH5, AT1G66530, MAT3, PPC2, At3g03780, GAPC1, G6PD5, HOG1, MTO2, CAC2, PB
GO:0019752	carboxylic acid metabolic process	2.02E-05	iPGAM1, SDH5, AT1G66530, PPC2, At3g03780, GAPC1, HOG1, MTO2, CAC2
GO:0006732	coenzyme metabolic process	3.68E-05	iPGAM1, MAT3, GAPC1, G6PD5, HOG1, CAC2
GO:0009266	response to temperature stimulus	5.18E-05	iPGAM1, GRP2B, GAPC1, RPN10, TSN1, RAD23D, PB
GO:0042221	response to chemical	5.74E-05	iPGAM1, SDH5, mtLPD1, PAE1, At3g03780, GAPC1, G6PD5, HSP23.6, RPN10, TSN1, RAN-1, RAN2, CLPC1
GO:0017144	drug metabolic process	1.70E-04	iPGAM1, SDH5, MAT3, PPC2, GAPC1, HOG1, PB
GO:0072521	purine-containing compound metabolic process	2.10E-04	iPGAM1, GAPC1, HOG1, CAC2, PB
GO:0055086	nucleobase-containing small molecule metabolic process	2.10E-04	iPGAM1, GAPC1, G6PD5, HOG1, CAC2, PB
GO:0019693	ribose phosphate metabolic process	2.50E-04	iPGAM1, GAPC1, G6PD5, CAC2, PB
GO:0050896	response to stimulus	4.00E-04	iPGAM1, SDH5, mtLPD1, PAE1, GRP2B, At3g03780, GAPC1, G6PD5, HSP23.6, RPN10, TSN1, RAN-1, RAN2, RAD23D, CLPC1, PB
GO:0006006	glucose metabolic process	4.00E-04	iPGAM1, GAPC1, G6PD5
GO:0009152	purine ribonucleotide biosynthetic process	5.60E-04	iPGAM1, GAPC1, CAC2, PB
GO:1901564	organonitrogen compound metabolic process	6.00E-04	iPGAM1, PAE1, AT1G66530, AT2G18110, At3g03780, GAPC1, G6PD5, HOG1, MTO2, RPN10, CAC2, RAD23D, CLPC1, PB
GO:0009117	nucleotide metabolic process	6.00E-04	iPGAM1, GAPC1, G6PD5, CAC2, PB
GO:1901566	organonitrogen compound biosynthetic process	6.60E-04	iPGAM1, AT1G66530, AT2G18110, At3g03780, GAPC1, MTO2, CAC2, PB
GO:0034655	nucleobase-containing compound catabolic process	6.60E-04	iPGAM1, GAPC1, HOG1, TSN1
GO:0009066	aspartate family amino acid metabolic process	7.50E-04	At3g03780, HOG1, MTO2
GO:0006091	generation of precursor metabolites and energy	7.50E-04	iPGAM1, SDH5, mtLPD1, PPC2, GAPC1
GO:0009628	response to abiotic stimulus	8.30E-04	iPGAM1, GRP2B, At3g03780, GAPC1, RPN10, TSN1, RAN-1, RAD23D, PB

GO:0046500	S-adenosylmethionine metabolic process	0.0011	MAT3, HOG1
GO:0009108	coenzyme biosynthetic process	0.0011	iPGAM1, MAT3, GAPC1, CAC2
GO:1901135	carbohydrate derivative metabolic process	0.0013	iPGAM1, GAPC1, G6PD5, HOG1, CAC2, PB
GO:0009150	purine ribonucleotide metabolic process	0.0013	iPGAM1, GAPC1, CAC2, PB
GO:0044237	cellular metabolic process	0.0016	iPGAM1, SDH5, mtLPD1, PAE1, AT1G66530, SUS6, AT2G18110, MAT3, PPC2, At3g03780, GAPC1, G6PD5, HOG1, MTO2, RPN10, TSN1, CAC2, RAD23D, PB
GO:0006754	ATP biosynthetic process	0.0016	iPGAM1, GAPC1, PB
GO:0000054	ribosomal subunit export from nucleus	0.0016	RAN-1, RAN2
GO:0071704	organic substance metabolic process	0.0018	iPGAM1, SDH5, PAE1, AT1G66530, SUS6, AT2G18110, MAT3, PPC2, At3g03780, GAPC1, G6PD5, HOG1, MTO2, RPN10, TSN1, CAC2, RAD23D, CLPC1, PB
GO:0046496	nicotinamide nucleotide metabolic process	0.0018	iPGAM1, GAPC1, G6PD5
GO:0046394	carboxylic acid biosynthetic process	0.0018	iPGAM1, At3g03780, GAPC1, MTO2, CAC2
GO:0009651	response to salt stress	0.0018	At3g03780, GAPC1, RPN10, TSN1, RAN-1
GO:0017038	protein import	0.0021	RAN-1, RAN2, CLPC1
GO:0009168	purine ribonucleoside monophosphate biosynthetic process	0.0021	iPGAM1, GAPC1, PB
GO:0008152	metabolic process	0.0021	iPGAM1, SDH5, mtLPD1, PAE1, AT1G66530, SUS6, AT2G18110, MAT3, PPC2, At3g03780, GAPC1, G6PD5, HOG1, MTO2, RPN10, TSN1, CAC2, RAD23D, CLPC1, PB
GO:0005488	binding	0.0023	iPGAM1, mtLPD1, AT1G66530, AT2G18110, GRP2B, MAT3, At3g03780, GAPC1, G6PD5, HOG1, MTO2, RPN10, TSN1, RAN-1, RAN2, CAC2, RAD23D, CLPC1, PB
GO:0044238	primary metabolic process	0.0025	iPGAM1, SDH5, PAE1, AT1G66530, SUS6, AT2G18110, PPC2, At3g03780, GAPC1, G6PD5, HOG1, MTO2, RPN10, TSN1, CAC2, RAD23D, CLPC1, PB
GO:0046034	ATP metabolic process	0.0027	iPGAM1, GAPC1, PB
GO:0044248	cellular catabolic process	0.0032	iPGAM1, PAE1, GAPC1, HOG1, RPN10, TSN1, RAD23D
GO:0006790	sulfur compound metabolic process	0.0032	MAT3, At3g03780, HOG1, CAC2
GO:0044272	sulfur compound biosynthetic process	0.0034	MAT3, At3g03780, CAC2
GO:1901575	organic substance catabolic process	0.0035	iPGAM1, PAE1, GAPC1, HOG1, RPN10, TSN1, RAD23D
GO:0009409	response to cold	0.0035	iPGAM1, GRP2B, RAD23D, PB
GO:0006730	one-carbon metabolic process	0.0035	MAT3, HOG1
GO:0009167	purine ribonucleoside monophosphate metabolic process	0.0036	iPGAM1, GAPC1, PB

GO:0006555	methionine metabolic process	0.0036	At3g03780, HOG1
GO:0006520	cellular amino acid metabolic process	0.0036	AT1G66530, At3g03780, HOG1, MTO2
GO:0072594	establishment of protein localization to organelle	0.0057	RAN-1, RAN2, CLPC1
GO:0009987	cellular process	0.0057	iPGAM1, SDH5, mtLPD1, PAE1, AT1G66530, SUS6, AT2G18110, MAT3, PPC2, At3g03780, GAPC1, G6PD5, HOG1, MTO2, RPN10, TSN1, CAC2, RAD23D, CLPC1, PB
GO:0009408	response to heat	0.0061	GAPC1, RPN10, TSN1
GO:0009067	aspartate family amino acid biosynthetic process	0.0061	At3g03780, MTO2
GO:0006606	protein import into nucleus	0.0064	RAN-1, RAN2
GO:0006101	citrate metabolic process	0.0078	SDH5, PPC2
GO:0006099	tricarboxylic acid cycle	0.0078	SDH5, PPC2
GO:0009735	response to cytokinin	0.0079	SDH5, RPN10, CLPC1
GO:0042866	pyruvate biosynthetic process	0.0092	iPGAM1, GAPC1
GO:0009744	response to sucrose	0.0092	GAPC1, RPN10
GO:0006757	ATP generation from ADP	0.0092	iPGAM1, GAPC1
GO:0006096	glycolytic process	0.0092	iPGAM1, GAPC1
GO:0000096	sulfur amino acid metabolic process	0.0092	At3g03780, HOG1
GO:0009166	nucleotide catabolic process	0.0093	iPGAM1, GAPC1
GO:0006725	cellular aromatic compound metabolic process	0.0093	iPGAM1, PAE1, AT1G66530, MAT3, GAPC1, G6PD5, HOG1, TSN1, CAC2, RAD23D, PB

Table S5. A heatmap comparing genus-level bacterial diversity and abundance among the controls and cold plasma treated groups based on high-throughput sequencing results. (presented in a separate MS Excel file)
